# Associations Between Sleep Duration and Sensory Impairments Among Older Adults in China

**DOI:** 10.3389/fnagi.2022.910231

**Published:** 2022-06-07

**Authors:** Hongguo Rong, Xiao Wang, Xiaozhen Lai, Weijie Yu, Yutong Fei

**Affiliations:** ^1^Institute for Excellence in Evidence-Based Chinese Medicine, Beijing University of Chinese Medicine, Beijing, China; ^2^Center for Evidence-Based Chinese Medicine, Beijing University of Chinese Medicine, Beijing, China; ^3^School of Traditional Chinese Medicine, Beijing University of Chinese Medicine, Beijing, China; ^4^Beijing Key Laboratory of Mental Disorders, The National Clinical Research Center for Mental Disorders, Anding Hospital, Capital Medical University, Beijing, China; ^5^China Center for Health Development Studies, Peking University, Beijing, China

**Keywords:** sleep duration, sensory function, visual impairment, hearing impairment, aging

## Abstract

**Objective:**

Studies of sleep duration in relation to the risk of sensory impairments other than dementia are scarce. Little is known about the associations between sleep duration and sensory impairments in China. This study aims to explore the associations between sleep duration and single or dual sensory impairments (visual and/or hearing).

**Methods:**

This cross-sectional study used the data from 17,668 respondents were drawn from the 2018 survey of the China Health and Retirement Longitudinal Study (CHARLS), an ongoing national longitudinal study of Chinese adults aged 45 years and above. The duration of sleep per night was obtained from face-to-face interviews. The presence of sensory impairments was measured by self-reported visual and hearing functions. Multivariable generalized linear models (GLM) with binomial family and log link to assess the associations between sleep duration and sensory impairments.

**Results:**

Of the 17,668 respondents, 8,396 (47.5%) were men. The mean (*SD*) age was 62.5 (10.0) years old. Respondents with short (≤ 4, 5 h per night) sleep duration had a significantly higher risk of visual, hearing and dual sensory impairments than those who slept for 7 h per night after adjusting for covariates (*P* < 0.05). Meanwhile, respondents who slept for 6 h per night had a higher risk of hearing impairment (*P* = 0.005). Further analysis suggested a U-shaped association between sleep duration and sensory impairments. When sleep duration fell below 8 h, increased sleep duration was associated with a significantly lower risk of visual (OR, 0.93; 95%CI, 0.88–0.98; *P* = 0.006), hearing (OR, 0.89; 95% CI, 0.86–0.93; *P* < 0.001), and dual (OR, 0.90; 95% CI, 0.87–0.94; *P* < 0.001) impairments. When sleep duration exceeded 8 h, the risk of visual (OR, 1.09; 95% CI, 1.00–1.19; *P* = 0.048), hearing (OR, 1.04; 95% CI, 0.97–1.11; *P* = 0.269), and dual (OR, 1.07; 95% CI, 1.00–1.14; *P* = 0.044) impairments would increase facing prolonged sleep duration. Women and the elderly aged over 60 years old were more sensitive to short sleep duration and experienced a higher risk of sensory impairments.

**Conclusion:**

In this study, short sleep duration was associated with a higher risk of visual and hearing impairments. Future studies are needed to examine the mechanisms of the associations between sleep duration and sensory impairments.

## Introduction

The elderly population has grown exponentially in recent decades worldwide and unexceptionally in China, the most populous country in the world. China is currently encountering the most important socioeconomic challenges brought about by the problem of aging. By 2050, it is estimated that China will have 400 million people aged 65 years and above, and 150 million aged 80 and above (Fang et al., 2015). Sensory impairments, typically impaired visual and hearing functions, are among the most common long-term conditions that are also associated with aging, placing an increasing burden on the healthcare system (Maharani et al., 2020). As estimated by the World Health Organization, at least 206 million people were experiencing some form of vision impairment globally in 2020 (GBD 2019 Blindness and Vision Impairment Collaborators and Vision Loss Expert Group of the Global Burden of Disease Study, 2021). It was also projected that nearly 2.5 billion people in the world would have some degree of hearing impairment by the year 2050 (Ratanjee-Vanmali et al., 2020).

Sleep duration is an important health-related factor in the development of physiological functions. It has been proven to be related with cognitive decline, depression, and other chronic problems (Ma et al., 2020; Matsui et al., 2020; Titova et al., 2021). However, findings on the associations between sleep duration and the risk of sensory impairments are still inconsistent. Evidence from the 2009 National Health Interview Survey (NHIS) in the United States showed that either scarce or excessive sleep was associated with visual impairment (Ramos et al., 2014). Of note, a cross-sectional study of 3,840 respondents aged 50 years or above in South Africa found that only a short sleep duration of less than 6 h was associated with self-reported visual impairment (Peltzer and Phaswana-Mafuya, 2017). Similarly, a study that used data from the baseline survey of the China Health and Retirement Longitudinal Study (CHARLS) suggested that short sleep duration was associated with a higher risk of visual impairment (Sun et al., 2021). As for hearing impairment, little is known about its associations with sleep duration. A study of 632 adults aged 70 years or above in the United States reported that longer sleep duration exceeding 8 h was linked with poorer high-frequency hearing (Jiang et al., 2021). Another study of 48,091 adults aged 20–79 years in Japan suggested that individuals with sleep duration exceeding 8 h had a higher risk of hearing loss (Nakajima et al., 2014). However, most previous studies only put insight into either visual or hearing impairment, instead of analyzing the two most crucial sensory functions under the same framework (Clark-Gambelunghe and Clark, 2015). Therefore, research on the associations between sleep duration and visual and/or hearing impairments is still lacking.

The prevalence rates of sensory impairments in China are higher in comparison to those in western countries, with a rapidly increasing aging population (Heine et al., 2019b). The 2013 CHARLS survey reported that the prevalence of visual, hearing and dual sensory impairments among residents aged 60 and over (*n* = 8268) was 80.2, 64.9, and 57.2%, respectively (Heine et al., 2019a). By contrast, the prevalence of visual impairment in Australia ranged from 4.37 to 46.15% for people aged 60 years and above (Heine et al., 2019a), and the prevalence of hearing loss in Western countries ranged from 45.5 to 100% with large variations by age (Cruickshanks et al., 2010). In addition, given the differences in historical, ethnic, economic, and socio-cultural background, the Chinese population has different lifestyles and sleep patterns compared to those living in western countries (Chen et al., 2014), and sleep insufficiency, insomnia and daytime sleepiness are more prevalent among Chinese adults (Liu et al., 2008). Therefore, this present study aims to explore the associations between short and long sleep duration and sensory impairments in China using a nationally representative sample of adults aged 45 years and above, and hypothesized that sleep duration is strongly linked with sensory impairments.

## Materials and Methods

**FIGURE 1:**
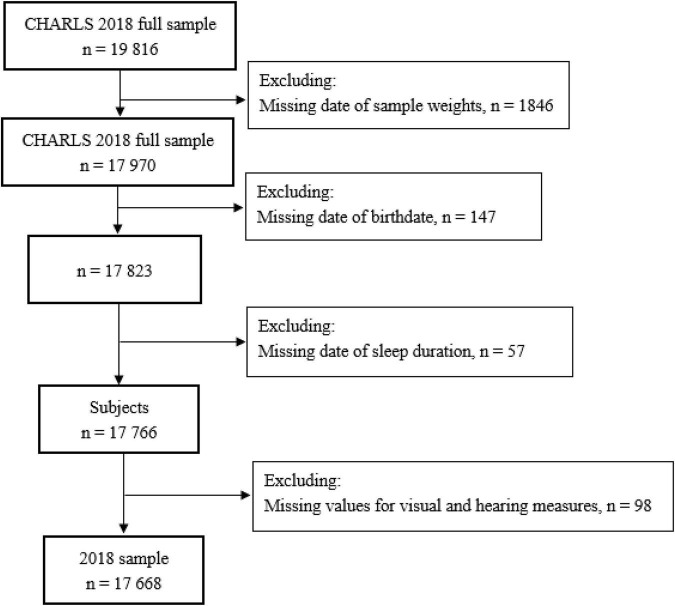
Flowchart of the study sample from the 2018 China Health and Retirement Longitudinal Study (CHARLS).

This study used data from the China Health and Retirement Longitudinal Study (CHARLS), an ongoing longitudinal study of Chinese adults aged 45 years or older and their spouses. Initiated in 2011, the CHARLS conducted follow-ups every 2 or 3 years in 2013, 2015, and 2018, to collect an excellent nationally representative sample of the middle-aged and elderly in China. The survey recruited respondents in 150 counties or districts and 450 villages or urban communities throughout the country using multistage stratified probability proportional to size sampling method. In the present study, we used the latest CHARLS wave in 2018 with 19,816 individuals to investigate the associations between sleep duration and sensory impairments. Figure 1 shows the schematic flow of the study sample. Our final sample included 17,668 individuals with or without sensory impairments.

The CHARLS protocol was in line with the Declaration of Helsinki (World Medical Association, 2013) and was ethically approved by the Peking University Institutional Review Board (Zhao et al., 2014). This cross-sectional study was conducted following the Strengthening the Reporting of Observational Studies in Epidemiology (STROBE) reporting guideline (von Elm et al., 2007). All participants in the CHARLS provided written informed consent.

### Sleep Duration

Similar to the English Longitudinal Study of Aging (ELSA) in England, CHARLS collected self-reported sleep duration without any given categories in face-to-face interviews. Sleep duration was assessed using the question: “In the past month, how many hours of sleep have you had on average for one night?” Consistent with a previous CHARLS study on sleep (Ma et al., 2020), we divided respondents into seven sleep duration groups (≤ 4, 5, 6, 7, 8, 9, and ≥ 10 h per night) in the analyses. Daytime napping was assessed by the question “In the past month, how long have you taken on average for a nap after lunch?” Four napping duration groups were set, including non-nappers (0 min), short nappers (< 30 min), moderate nappers (30–90 min), and extended nappers (> 90 min).

### Sensory Functions

Sensory impairments were identified using self-reported assessments of visual and hearing functions. Similar to the Health and Retirement Study (HRS) in the United States, CHARLS collected self-reported data on visual functions using two questions: “Is your eyesight for seeing things at a distance excellent (1), very good (2), good (3), fair (4), or poor (5)?” and “How good is your eyesight for seeing things up close, like reading ordinary newspaper print? Would you say your eyesight for seeing things up close is excellent (1), very good (2), good (3), fair (4), or poor (5)?” Data on hearing functions were also collected using the question: “Is your hearing excellent (1), very good (2), good (3), fair (4), or poor (5)?” Respondents were recorded as having visual or hearing impairment if they reported fair or poor vision (for either long-distance or near vision) or hearing (Whillans and Nazroo, 2014; Maharani et al., 2018).

### Covariates

The CHARLS structured questionnaire conducted face-to-face interviews to collect respondents’ demographic characteristics, health habits, and clinical characteristics. Covariates that might confound the associations between sleep duration and sensory impairments in the analyses included age (in years), gender (men vs. women), education level (i.e., illiterate, primary or middle school, and high school or above), tobacco use (never vs. current), alcohol use (never vs. current), marital status (married or cohabited vs. otherwise), afternoon napping, residence (urban vs. rural), depressive symptoms, activities of daily living (ADLs), chronic disease condition (i.e., none, mild, and severe), and sampling weights. The ADLs in CHARLS were measured using a 6-item summary assessed with an ADL scale that includes eating, dressing, transferring, bathing, using the toilet, and continence. The respondents were considered independent if they were able to complete all 6 activities without difficulty. The 10-item Center for Epidemiological Studies Depression Scale (CES-D-10) was used to estimate respondents’ depressive symptoms, with a possible score ranging from 0 to 30 (Cronbach α = 0.815) (Lei et al., 1982). Previous studies suggested a cutoff point greater than or equal to 10 to differentiate between those with and without depression (Luo et al., 2018; Zhou et al., 2021). Respondents’ chronic disease condition was captured based on the number of self-reported non-communicable diseases (NCDs), including hypertension, diabetes, dyslipidemia, heart problems, stroke, kidney diseases, asthma, lung diseases, arthritis, liver diseases, and stomach diseases. Similar to a previous CHARLS study on sleep duration (Sun et al., 2018), we categorized individuals into three chronic disease groups “none” (no NCD), “mild” (1–2 types of NCDs) or “severe” (more than three types of NCDs). As for sampling weights, we adopted the sets of cross-sectional individual weights with corrections for household and individual non-response corrections.

### Statistical Analysis

In the present study, respondents’ general characteristics were compared by sensory impairment status (visual impairment, hearing impairment, and dual sensory impairments) using an analysis of variance for numerical variables and ordinal χ^2^ tests for discrete variables. Continuous variables were reported using means and SDs. Categorical variables were presented using numbers and percentages. We adopted multivariable generalized linear models (GLM) with binomial family and log links to assess the associations between sleep duration and sensory impairments. We also used restricted cubic splines with four knots at the 5th, 35th, 65th, and 95th centiles to flexibly model the association of sleep duration with sensory impairments. Analyses were first unadjusted and then fully adjusted for other confounding factors. Age, gender, education, marital status, tobacco and alcohol use, afternoon napping, place of residence (urban or rural), depressive symptoms (based on CES-D-10), independence (based on ADLs), chronic disease condition (the number of NCDs), and sampling weights were included in our analytic models as covariates. Multiple imputation was adopted to deal with missing data. We also conducted analyses stratified by gender and age to investigate gender- or age-specific associations. We considered a two-sided *P* < 0.05 as statistically significant. All data analyses were conducted using Stata version 14.0 (StataCorp).

## Results

### Sample Characteristics

The characteristics of respondents in the 2018 CHARLS wave were shown in Table 1. The study sample consisted of 17,668 respondents (8,396 men and 9,272 women), with a mean age of 62.5 years. A total of 14,590 (82.6%) reported visual impairment, 11,688 (66.2%) reported hearing impairment, and 10,873 (61.5%) reported dual impairments. Compared with respondents without any sensory impairments, those who reported one or more sensory impairments had fewer hours of sleep per night on average (no visual impairment vs. visual impairment: 6.6 vs. 6.1; no hearing impairment vs. hearing impairment 6.5 vs. 6.1; no dual sensory impairments vs. dual sensory impairments 6.5 vs. 6.1). This sample reported a mean sleep duration of 6.2 h and the majority of respondents had their sleep duration between 6 and 8 h. Single or dual impairments were more prevalent among women, non-smokers, current alcohol users, the unmarried, rural residents, as well as those with lower education level, depression, impaired ADLs, and more chronic diseases.

**TABLE 1:** Characteristics of participants from 2018 China Health And Retirement Longitudinal Study, by sensory impairment status^a^.

Characteristics	Participants, no. (%)						
	Total sample (*N* = 17,668)	Visual impairment		Hearing impairment		Dual sensory impairment	
		Yes (*n* = 14,590)	No (*n* = 3,078)	Yes (*n* = 11,688)	No (*n* = 5,980)	Yes (*n* = 10,873)	No (*n* = 6,795)
Sleep duration per night, hours, mean ± *SD*	6.2 ± 2.0	6.1 ± 2.1	6.6 ± 1.9	6.1 ± 2.1	6.5 ± 1.8	6.1 ± 2.1	6.5 ± 1.9
**Sleep duration per night in hours, *n* (%)**							
≤4	3,177 (18.0)	2,818 (19.3)	359 (11.7)	2,421 (20.7)	756 (12.8)	2,309 (21.2)	868 (12.8)
5	2,606 (14.8)	2,234 (15.3)	372 (12.1)	1,843 (15.8)	763 (12.8)	1,713 (15.8)	893 (13.1)
6	3,863 (21.9)	3,188 (21.9)	675 (21.9)	2,514 (21.5)	1,349 (22.6)	2,335 (21.5)	1,528 (22.5)
7	2,974 (16.8)	2,367 (16.2)	607 (19.7)	1,764 (15.1)	1,210 (20.2)	1,640 (15.1)	1,334 (19.6)
8	3,339 (18.9)	2,595 (17.8)	744 (24.2)	2,032 (17.4)	1,307 (21.9)	1,838 (16.9)	1,501 (22.1)
9	812 (4.6)	653 (4.5)	159 (5.2)	521 (4.5)	291 (4.9)	481 (4.4)	331 (4.9)
≥10	897 (5.1)	735 (5.0)	162 (5.3)	593 (5.1)	304 (5.1)	557 (5.1)	340 (5.0)
Age, mean (*SD*), y	62.5 (10.0)	62.9 (10.0)	60.9 (9.9)	63.7 (10.1)	60.3 (9.5)	63.7 (10.1)	60.7 (9.7)
**Gender, *n* (%)**							
Men	8,396 (47.5)	6,711 (46.0)	1,685 (54.7)	5,507 (47.1)	2,889 (48.3)	5,037 (46.3)	3,359 (49.4)
Women	9,272 (52.5)	7,879 (54.0)	1,393 (45.3)	6,181 (52.3)	3,091 (51.7)	5,836 (53.7)	3,436 (50.6)
**Education, *n* (%)**							
Illiterate	4,140 (25.6)	3,537 (24.2)	603 (19.6)	2,963 (25.4)	1,177 (19.7)	2,783 (25.6)	1,357 (20.0)
Primary or middle school	11,403 (64.5)	9,507 (65.2)	1,896 (61.6)	7643 (65.4)	3,760 (62.9)	7,139 (65.7)	4,264 (62.8)
High school or above	2,125 (12.0)	1,546 (10.6)	579 (18.8)	1,082 (9.3)	1,043 (17.4)	951 (8.8)	1,174 (17.3)
**Tobacco use, *n* (%)**							
Never	10,127 (57.9)	8,451 (58.5)	1,676 (55.1)	6,652 (57.6)	3,475 (58.7)	6,240 (58.0)	3,887 (57.8)
Current	7,354 (41.5)	5,988 (41.5)	1,366 (44.9)	4,906 (42.5)	2,448 (41.3)	4,517 (42.0)	2,837 (42.2)
**Alcohol use, *n* (%)**							
Never	11,474 (65.0)	9,638 (66.1)	1,836 (59.7)	3,945 (33.8)	3,733 (62.4)	7,241 (66.6)	4,233 (62.3)
Current	6,192 (35.1)	4,950 (33.9)	1,242 (40.4)	2,247 (38.6)	2,247 (37.6)	3,630 (33.4)	2,562 (37.7)
Married or cohabited, *n* (%)	13,921 (78.8)	11,455 (78.5)	2,466 (80.1)	9,085 (77.7)	4,836 (80.9)	8,460 (77.8)	5,461 (80.4)
**Residence, *n* (%)**							
Rural	14,040 (79.7)	11,792 (81.0)	2,248 (73.5)	9,568 (82.0)	4,472 (75.1)	8,974 (82.7)	5,066 (74.9)
Urban	3,585 (20.3)	2,773 (19.0)	812 (26.5)	2,102 (18.0)	1,483 (24.9)	1,883 (17.3)	1,702 (25.2)
Depression^b^, *n* (%)	6,169 (34.9)	5,554 (38.1)	615 (20.0)	4,664 (39.9)	1,505 (25.2)	4,459 (41.0)	1,710 (25.2)
ADLs impaired, *n* (%)	3,510 (18.1)	3,023 (20.9)	282 (9.4)	2,664 (22.9)	641 (10.9)	2,553 (23.6)	752 (11.3)
**Daytime napping in minutes, *n* (%)**							
None	6,755 (38.2)	5,640 (38.7)	1,115 (36.2)	4,503 (38.5)	2,252 (37.7)	4,160 (38.3)	2,714 (39.9)
≤30	1,467 (8.3)	1,184 (8.1)	283 (9.2)	954 (8.2)	513 (8.6)	882 (8.1)	585 (8.6)
31–90	6,874 (39.1)	5,659 (38.8)	1,215 (39.5)	4,466 (38.2)	2,408 (40.3)	4,160 (38.3)	2,714 (39.9)
≥90	2,572 (14.6)	2,107 (14.4)	465 (15.1)	1,765 (15.1)	807 (13.5)	1,629 (15.0)	943 (13.9)
**Chronic disease condition, *n* (%)**							
None	9,923 (56.2)	7,971 (54.6)	1,952 (63.4)	6,267 (53.6)	3,656 (61.1)	5,807 (53.4)	4,116 (60.6)
Mild	6,701 (37.9)	5,698 (39.1)	1,003 (32.6)	4,646 (39.8)	2,055 (34.5)	4,334 (39.9)	2,367 (34.8)
Severe	1,044 (5.9)	921 (6.3)	123 (4.0)	775 (6.6)	269 (4.5)	732 (6.7)	312 (4.6)

*ADLs, activities of daily living.*

*^a^Missing data for the following characteristics: tobacco use (187 [1.1%]), alcohol use (2 [ < 0.1%]), residence (43 [0.2%]), and ADLs impaired (158 [0.9%]).*

*^b^Defined as a score of 10 or greater on the 10-item Center for Epidemiologic Studies Depression scale.*

### Trajectories for Sleep Duration Across Sensory Impairment Status

**FIGURE 2:**
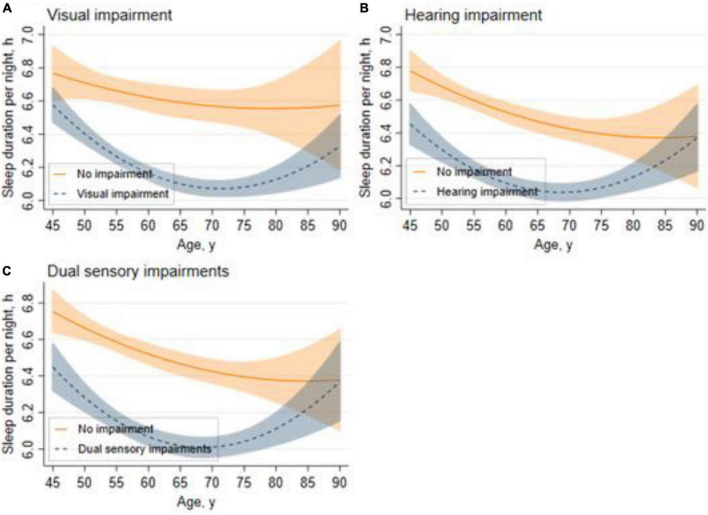
Trajectories for sleep duration across different sensory impairment groups. Graphs display analog values (lines) of sleep duration with 95% CIs (shaded areas) for visual impairment **(A)**, hearing impairment **(B)**, and dual sensory impairments **(C)**.

Figure 2 shows the trajectories for sleep duration across different sensory impairment groups. In general, the sleep duration of adults aged 45 and above in China took a shape of curvilinear trajectories. Figure 2A shows that for respondents with visual impairment, the sleep duration trajectories took a U-shape with age, and the shortest sleep duration happened at around the age of 70 years. Similar patterns are illustrated in Figure 2B: respondents with hearing impairment showed a rapid decline in sleep duration between the ages of 45–70 years, and increased after the age of 70. The sleep duration of respondents with no sensory impairment followed curvilinear shapes, while there was still a U-shaped association between sleep duration and age among respondents with dual sensory impairments (Figure 2C).

### Associations Between Sleep Duration and Sensory Impairments

Table 2 shows the relationships between sleep duration and sensory impairments, the unadjusted model (Model 1) showed that individuals reporting short (≤ 4, 5, 6 h) or long (9, ≥ 10 h) sleep duration per night had a significantly higher risk of hearing and dual sensory impairments than the reference group (7 h) (All *p* < 0.05). Similarly, respondents reporting shorter (≤ 4, 5, 6 h) sleep duration had significantly higher odds of visual impairment than the reference group (All *p* < 0.01). After adjusting for age, gender and other potential confounders, the associations between short (≤ 4, 5 h) sleep duration and different types of sensory impairments remained the same, while those between longer (9, ≥ 10 h) sleep duration and hearing or dual sensory impairments disappeared. Additionally, individuals reporting 6 h of sleep per night tended to have a significantly higher risk of hearing impairment [odds ratio (OR), 1.24; 95% confidence interval (CI), 1.01–1.44].

**TABLE 2:** Associations between sleep duration and sensory impairments in participants from the 2018 China Health and Retirement Longitudinal Study.

Sleep duration per night, h	Visual impairment		Hearing impairment		Dual sensory impairments	
	OR (95% CI)	*P*-value	OR (95% CI)	*P*-value	OR (95% CI)	*P*-value
**Model 1^a^**						
≤4	2.01 (1.74–2.32)	<0.001	2.20 (1.97–2.45)	<0.001	2.16 (1.95–2.41)	<0.001
5	1.54 (1.34–1.77)	<0.001	1.66 (1.48–1.85)	<0.001	1.56 (1.40–1.74)	<0.001
6	1.21 (1.07–1.37)	0.002	1.28 (1.16–1.41)	<0.001	1.24 (1.13–1.37)	<0.001
7	[Reference]	NA	[Reference]	NA	[Reference]	NA
8	0.89 (0.79–1.01)	0.070	1.07 (0.96–1.18)	0.212	1.00 (0.90–1.10)	0.938
9	1.05 (0.87–1.28)	0.602	1.23 (1.05–1.44)	0.012	1.18 (1.01–1.38)	0.037
≥10	1.16 (0.96–1.41)	0.122	1.34 (1.14–1.56)	<0.001	1.33 (1.14–1.55)	<0.001
**Model 2^b^**						
≤4	1.40 (1.14–1.71)	0.001	1.53 (1.31–1.80)	<0.001	1.53 (1.31–1.79)	<0.001
5	1.28 (1.02–1.61)	0.030	1.60 (1.34–1.90)	<0.001	1.38 (1.16–1.64)	<0.001
6	1.08 (0.89–1.31)	0.413	1.24 (1.07–1.44)	0.005	1.16 (1.00–1.34)	0.050
7	[Reference]	NA	[Reference]	NA	[Reference]	NA
8	0.84 (0.70–1.00)	0.050	0.98 (0.85–1.13)	0.791	0.91 (0.79–1.04)	0.170
9	0.94 (0.72–1.22)	0.629	1.09 (0.89–1.35)	0.400	1.02 (0.83–1.26)	0.815
≥10	1.10 (0.85–1.43)	0.453	1.02 (0.82–1.27)	0.861	1.07 (0.87–1.32)	0.540

*OR, odds ratio; CI, confidence interval.*

*^a^Model 1 was unadjusted.*

*^b^Model 2 was adjusted for age, gender, education, marital status, tobacco use, alcohol use, afternoon napping, residence, activities of daily living, depression, chronic diseases conditions, and sampling weights.*

### Sensitivity Analyses

**FIGURE 3:**
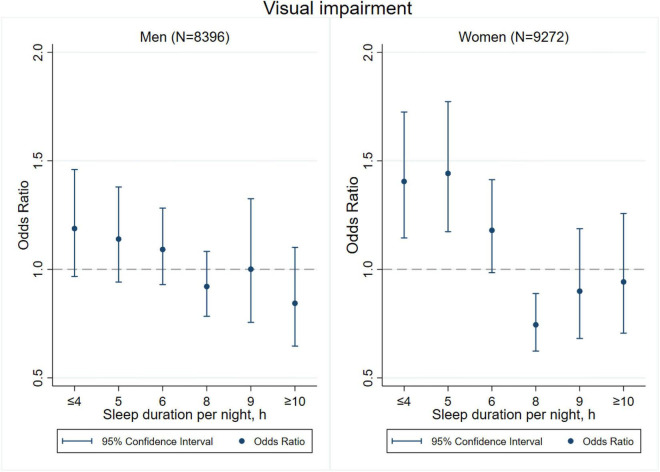
Gender-specific effect of sleep duration on visual impairment.

**FIGURE 4:**
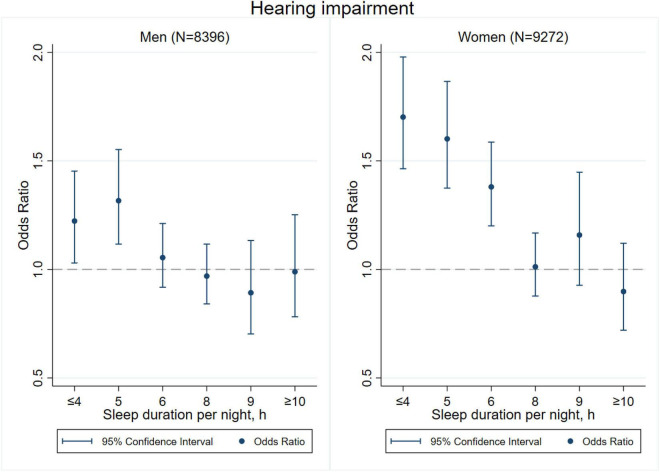
Gender-specific effect of sleep duration on hearing impairment.

**FIGURE 5:**
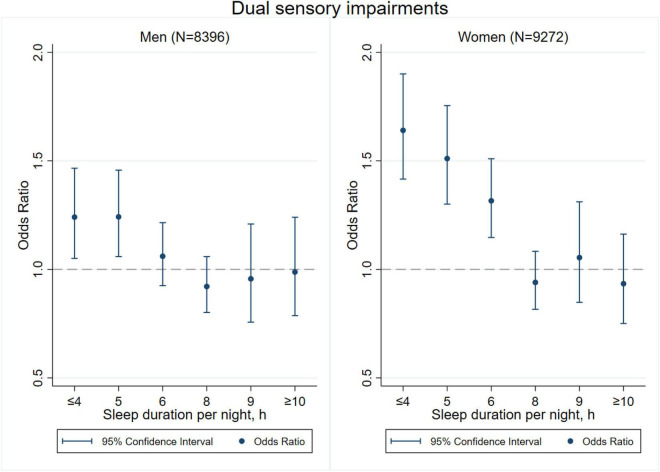
Gender-specific effect of sleep duration on dual sensory impairments.

**FIGURE 6:**
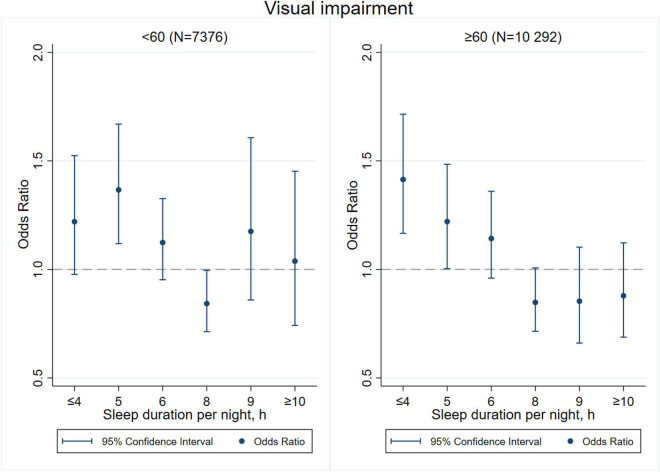
Age-specific effect of sleep duration on visual impairment.

**FIGURE 7:**
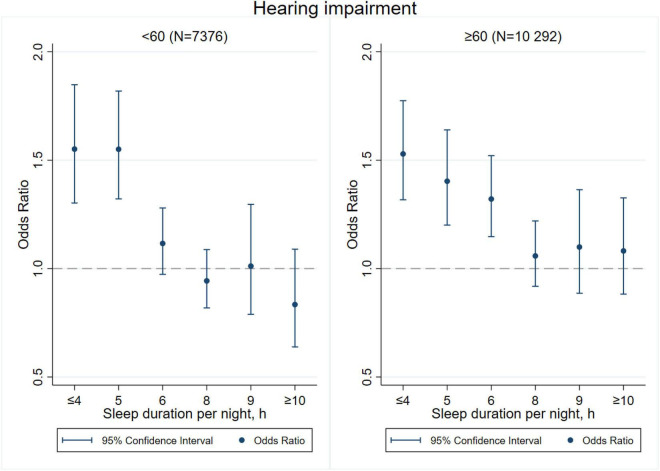
Age-specific effect of sleep duration on hearing impairment.

**FIGURE 8:**
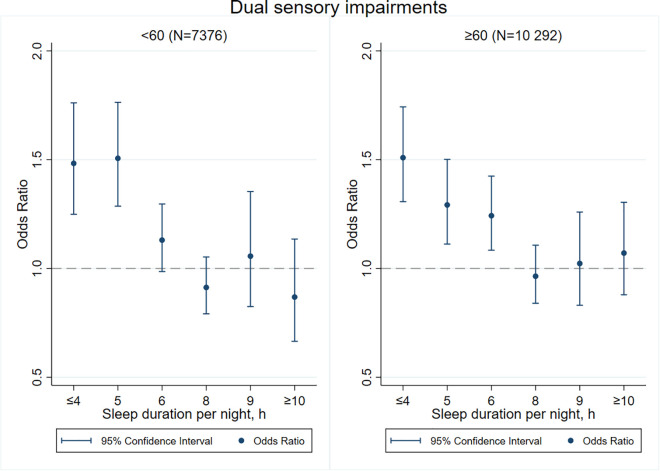
Age-specific effect of sleep duration on dual sensory impairments.

Figures 3–8 provide the results of stratified analysis by gender and age, in which significant gender- and age-specific effects were observed. Women with shorter sleep duration (≤ 4, 5, 6 h) had a higher risk of visual (Figure 3), hearing (Figure 4), and dual sensory impairments (Figure 5) compared to men. As for age-specific effects, the elderly aged 60 years and above with shorter sleep duration of less than 6 h experienced a higher risk of visual (Figure 6), hearing (Figure 7), and dual sensory impairments (Figure 8) than those aged under 60 years.

### Non-linear Relationship Between Sleep Duration and Sensory Impairments

**FIGURE 9:**
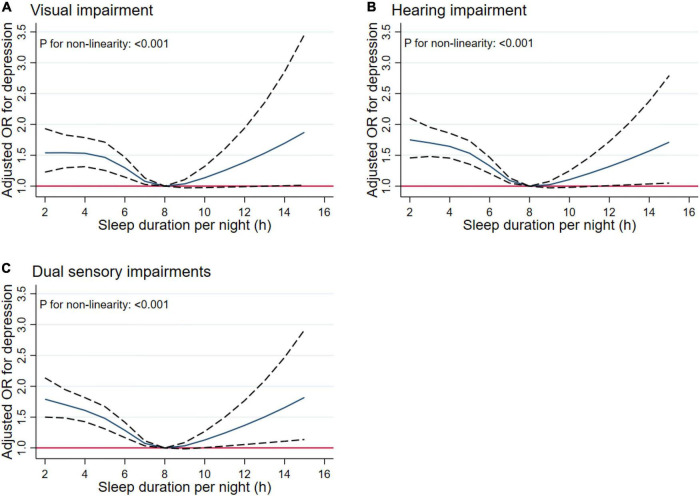
Non-linear relationship of sleep duration and sensory impairments. Graphs display adjusted OR for depression (solid lines) of sleep duration with 95% CIs (dotted lines) for visual impairment **(A)**, hearing impairment **(B)**, and dual sensory impairments **(C)**.

In Figure 9, we used restricted cubic splines to flexibly model and visualize the relations between predicted sleep duration and sensory impairments. Figure 9 presents similar U-shaped associations between sleep duration and visual, hearing and dual sensory impairments after adjusting for potential confounding factors. As Figure 9A shows, the risk of visual impairment was negatively correlated with sleep duration until it bottomed out at 8 h (OR, 0.93; 95% CI, 0.88–0.98; *P* = 0.006). However, when the sleep duration was higher than 8 h, the risk of hearing impairment increased significantly (OR, 1.09; 95% CI, 1.00–1.19; *P* = 0.048). As Figure 9B indicates, the risk of hearing impairment decreased until 8 h of predicted sleep duration, and then started to increase rapidly afterward (*P* for non-linearity < 0.001). Regarding the strong U shaped relation between sleep duration and dual sensory impairments, Figure 9C demonstrates a substantial reduction in the risk of dual sensory impairments within the lower range of predicted sleep duration, which reached the lowest risk at around 8 h and increased thereafter (*P* for non-linearity < 0.001). Below 8 h, the adjusted OR per standard deviation higher predicted sleep duration was 0.90 (95% CI, 0.87–0.94; *P* < 0.001). Above 8 h, the adjusted OR per standard deviation higher predicted sleep duration was 1.07 (95% CI, 1.00–1.14; *P* = 0.044).

## Discussion

Our findings derived from the nationally representative sample of 19,369 adults aged 45 years and older in China reported that shorter sleep duration was associated with a higher risk of self-reported visual, hearing and dual sensory impairments. We also found that women and individuals aged 60 years and above were more sensitive to the associations between short sleep duration and sensory impairments. However, we did not observe a similar association between long sleep duration and sensory impairments. To the best of our knowledge, the present study is among the largest-scale investigations of the relationships between sleep duration and sensory impairments in China.

The prevalence rates of visual and hearing impairment among older Chinese adults were higher in comparison to those in Western Europe (Heine et al., 2019a). As individuals age, disrupted sleep patterns are associated with remarkable brain changes, worsening sensory processing and quality of life (Li et al., 2018), so higher prevalence rates of sensory impairments were observed among older adults. Research on the associations between sleep duration and visual and/or hearing impairments is still lacking. Our previous study suggested that sensory impairments were significantly associated with cognitive decline (Rong et al., 2020). A number of epidemiological studies reported a U-shaped association between sleep duration and cognitive decline (Chen et al., 2016; Ma et al., 2020; Joo et al., 2021), indicating the effects of both short and long sleep duration on cognitive aging. What are the associations between sleep duration and sensory impairments in the general aging population, and is there also a U-shaped relationship? It is important to explore whether sensory impairments should be monitored in individuals with insufficient or excessive sleep duration. Compelling evidence suggested that sleep duration may be involved in the development of sensory impairments. However, a consistent conclusion has not yet been fully achieved.

Previous studies also investigated the associations between sleep duration and sensory functions, however, most of them only focused on one type of sensory impairment (Ramos et al., 2014; Ghemrawi et al., 2021). Peltzer and Phaswana-Mafuya (2017) analyzed data from the Study of Global Aging and Adults Health (SAGE) wave 1 in 2008 and noted that individuals with short sleep duration (≤ 5 h per day) had a higher risk of self-reported visual impairment. A case-control study using pooled controls in Japan reported that short sleep duration might be a risk factor for idiopathic sudden deafness (Nakamura et al., 2001). The 2005–2006 cycle of the National Health and Nutrition Examination Survey (NHANES) in the United States suggested that longer sleep duration (≥ 8 h per day) was marginally associated with poorer hearing function (Jiang et al., 2021). However, this study was weak in representativeness due to the relatively small sample size of fewer than 1,000 individuals (632 older adults aged 70 years and older). Our study specially investigated the associations between sleep duration and dual sensory impairment (both visual and hearing), indicated that individuals with short sleep duration had a higher risk of visual, hearing, and dual sensory impairments, consistent with the results of previous studies (Ramos et al., 2014; Ghemrawi et al., 2021). Nevertheless, our results did not find out any significant association between long sleep duration and sensory impairment. The link between sleep duration and sensory impairment is complex, and the effects of long sleep duration remain uncertain, although a systematic review of observational studies reported that long sleep duration was associated with an increased risk of neurodegenerative disease (Devore et al., 2016). Therefore, we should be cautious about the generalizability of the findings of this study.

The mechanism that how sleep duration regulates sensory impairments has not been fully explained. There are several hypothesized mechanisms of the adverse effects of short sleep duration on sensory impairments. One possible explanation is that, as proposed by prior studies on sleep and brain structure, lower cortical volumes predominantly in frontal and parietal regions caused by short sleep duration was associated with hearing impairment (Brenowitz et al., 2021; Jalbrzikowski et al., 2021). Additionally, short sleep duration can give rise to chronic, systemic low-grade inflammation by impairing the hypothalamic-pituitary-adrenal (HPA) axis which is crucial in suppressing inflammatory process, so it is related to various diseases derived from disturbed metabolic clearance, such as diabetes, hypertension, and neurodegeneration (Lu et al., 2015; Ogilvie and Patel, 2018; Choe et al., 2019). Besides, sleep deterioration can occur as a result of several diseases or symptoms that can also cause visual impairment (Asplund, 2000). For example, people who suffer from vascular disease or diabetes have an increased risk of sleep disorders (Cappuccio and Miller, 2017), while vascular disease or diabetes are also known as common causes of visual impairment (Petrella et al., 2012). Visual impairment results in lower exposure to ambient illumination, causing a subsequent reduction in melatonin secreted from the pineal gland (Jean-Louis et al., 2005). Reduced melatonin levels can potentially lead to increased depressive symptoms, which are linked to insomnia (Taylor et al., 2005). As expected, the results in the current study also pointed out that respondents with single or dual sensory impairments had higher prevalence of diabetes, hypertension, and other cardiovascular diseases.

Our stratified analyses suggested that women and the elderly aged 60 years and above with shorter sleep duration (≤ 4, 5, 6 h) had a higher risk of visual, hearing, and dual sensory impairments. Gender differences in sleep duration among the aged population cannot be ignored as insomnia is generally higher in women (Romanella et al., 2021). Our findings were in concordance with the United States National Institute on Aging’s multicentered study entitled “Established Populations for Epidemiologic Studies of the Elderly” (EPESE) (Foley et al., 1995) and the Spanish National Health Survey 2017 (Pardhan et al., 2021), which found that women were more likely to sleep less or have trouble in sleeping, and had a higher risk of visual, hearing and dual sensory impairments than men. One possible explanation is that women may adapt more easily to the health burden, including depression, than their male counterparts (Schechtman et al., 1997), and therefore, they may be less likely to be extreme sleepers. Another reason may be related to dietary intake. As for age-specific effects, changes in sleep patterns (including sleep duration) are a part of the normal aging process. Sleep duration decreases with aging, and older people tend to have difficulty in falling and staying asleep. As a result, sleep debt increases with age (Chen et al., 2014). According to the reports on middle-aged Chinese population, increased pain, cardiovascular diseases, and neurological disorders were found in those sleeping less than 4–5 h per night (Gulia and Kumar, 2018; Lao et al., 2018; Ma et al., 2020). Elderly people spend less time in a deeper stage of sleep, and short sleep duration increases the risk of visual impairment, hearing impairment, and dual sensory impairments among the elderly. The results emphasize on the importance of addressing the complex needs of sensory impaired population, particularly among women and the elderly with short sleep duration.

## Limitations

There were also some limitations in this study. First, only self-reported sleep duration and sensory impairments were assessed without objective assessments of the quality of sleep, sleep disturbance, difficulties in falling asleep, and severity of sensory impairments, which could be subject to potential biases. Second, similar to most other epidemiological studies on sensory impairments, the use of self-reported measures to capture sensory functions might underestimate the associations between sleep duration and sensory impairments. Finally, the cross-sectional nature of this study prevents inferences about causal relationships. Short sleep duration might be an early manifestation of brain impairment, and whether short sleep duration is a marker or a risk factor of sensory impairments remains unclear.

## Conclusion

In conclusion, the associations between sleep duration and sensory impairments were investigated using a nationally representative sample in China. The findings suggested that short sleep duration was associated with higher risks of visual, hearing, and dual sensory impairments. Women and those aged 60 years and above were more sensitive to the associations between short sleep duration and sensory impairments. Further studies investigating the mechanism of such associations are need.

## Data Availability Statement

The datasets presented in this study can be found in online repositories. The names of the repository/repositories and accession number(s) can be found below: https://charls.pku.edu.cn.

## Ethics Statement

The studies involving human participants were reviewed and approved by the Peking University Institutional Review Board. The patients/participants provided their written informed consent to participate in this study.

## Author Contributions

YF had full access to all of the data in the study and takes responsibility for the integrity of the data and the accuracy of the data analysis. HR, XW, XL, WY, and YF contributed to the hypothesis and study design and interpreted the result. HR, XW, and XL analyzed the data. HR and XL wrote the manuscript. All authors contributed to the article and approved the submitted version.

## Conflict of Interest

The authors declare that the research was conducted in the absence of any commercial or financial relationships that could be construed as a potential conflict of interest.

## Publisher’s Note

All claims expressed in this article are solely those of the authors and do not necessarily represent those of their affiliated organizations, or those of the publisher, the editors and the reviewers. Any product that may be evaluated in this article, or claim that may be made by its manufacturer, is not guaranteed or endorsed by the publisher.
